# Postural Effects of Vestibular Manipulation Depend on the Physical Activity Status

**DOI:** 10.1371/journal.pone.0162966

**Published:** 2016-09-14

**Authors:** Julien Maitre, Thierry Paillard

**Affiliations:** Laboratoire Mouvement, Equilibre, Performance et Santé, EA 4445, Université de Pau et des Pays de l’Adour, Département STAPS, Tarbes, France; McGill University Department of Physiology, CANADA

## Abstract

The purpose of this study was to compare the effects of galvanic vestibular stimulation (GVS) on postural control for participants of different physical activity status (i.e. active and non-active). Two groups of participants were recruited: one group of participants who regularly practised sports activities (active group, n = 17), and one group of participants who did not practise physical and/or sports activities (non-active group, n = 17). They were compared in a reference condition (i.e bipedal stance with eyes open) and four vestibular manipulation condition (i.e. GVS at 0.5 mA and 3 mA, in accordance with two designs) lasting 20 seconds. The centre of foot pressure displacement velocities were compared between the two groups. The main results indicate that the regular practice of sports activities counteracts postural control disruption caused by GVS. The active group demonstrated better postural control than the non-active group when subjected to higher vestibular manipulation. The active group may have developed their ability to reduce the influence of inaccurate vestibular signals. The active participants could identify the relevant sensory input, thought a better central integration, which enables them to switch faster between sensory inputs.

## Introduction

The vestibular system encodes self-motion information by detecting the position and the motion of the head in space (i.e. angular and linear accelerations), which contributes to the control of eyes movements and stabilisation of the gaze, the perception of body orientation, the control of posture and balance [1–4]. The central nervous system integrates the vestibular afferents and afferents from other sensory systems to maintain an upright stance [5,6]. The vestibular afferent signal content may influence the reliance attributed to other sensory information [7,8]. Alteration in how the central nervous system (CNS) integrates vestibular afferents with other sensory information can lead to impaired movement coordination, vertigo, spatial disorientation, and perceptual illusions [9]. Furthermore, the ability to integrate vestibular afferents constitutes a major factor of efficiency in airplane flight and sport activities, which may have serious safety implications for airplane pilots and sportsmen [10,11]. Indeed, head displacements during airplane flight and sport activities may elicit acute vestibular illusion of motion (i.e. erroneous perception of head motion), generating sensory conflicts between visual and vestibular systems, which results in spatial disorientation [12–14]. The vestibular illusion of motion may refer to the sensation of motion, comprising both spinning and linear movements, when none exists, linked to the endolymph and the macula inertia during head movements. Otherwise, in a context of a pathological alteration of the vestibular structure, individuals may experiment vestibular illusion of motion. For example, in benign paroxysmal positional vertigo, otoconia can become dissociated from the otolithic membrane and released in the semicircular canal ampullae system as free-floating particles (canalithiasis theory) or fixed particles on the cupula (cupulolithiasis theory) [15–19], which may alter vestibular function [20,21].

When vestibular afferents are altered the CNS triggers mechanisms to compensate the alteration [22]. Vestibular adaptation mainly consists in the capability of the vestibular system to modulate the magnitude of the vestibulo-ocular reflex (VOR), depending on a specific context (e.g., orientation of the head, position of the eye in the orbit) to help the stabilization of vision [22–25]. Otherwise, repetitive exposure to a vestibular stimulation (e.g. acceleration and deceleration) enable a long lasting phenomenon called habituation [10,12,13] characterized by a progressive reduction of the intensity of the stimulus response limited to the characteristics of the stimulus (e.g., direction and plane) and a decrease of the duration of the stimulus perception [13,22,26,27]. The ability to use correctly vestibular and visual cues by the use of the adaption and habituation mechanisms contributes to human ego-motion perception during airplane flight and sport activities [28]. A decrease in the duration of vestibular illusion of motion may serve to alleviate the disorientating consequences of visuo-vestibular conflict [13,29] during flight or sport activities.

In a context where vestibular system is disrupted, postural control can be impaired [30]. When a sensory modality is altered (e.g. sensory perturbation), the CNS triggers compensatory postural strategies, sensory reweighting and feed-forward mechanisms to preserve postural control [5,6,31]. The regular practice of physical and sports activities may improve these mechanisms [32,33,34]. Furthermore, it may improve sensory sensitivity and develop the specific ability to switch from one sensory channel to another sensory channel that is better adapted to the postural conditions induced by the perturbation [33,34]. In addition, physical activity may improve the perception of body orientation [35]. These mechanisms improve postural abilities and thereby may reduce the postural effects of a sensory disruption [31].

Galvanic vestibular stimulation (GVS) has been widely used as a sensory manipulation to alter vestibular afferents signal in order to understand how an individual counteracts the postural effects of the artificial vestibular stimuli [30,36,37]. The GVS technique generates an electrical current, which is delivered transcutaneously to the vestibular system through electrodes placed over the mastoid bones. This technique modulates the firing level of vestibular afferents (i.e., cathodal current depolarize and thus increase the firing rate of vestibular afferents whereas anodal current hyperpolarize and decrease their firing rate) and so produces a signal of head movement that has a potent effect on whole body motor control. This fictitious vestibular signal provokes an illusion of head motion and a well-organised postural response of the whole body depending on the GVS design, the current intensity, the postural task and the availability of other sensory information [30]. GVS enables several designs (e.g., monaural or binaural stimulation with a monopolar or a bipolar polarity) [30,36–39]. On the one hand, when the head facing forward, a binaural bipolar GVS induces body sways in the frontal plan in the direction of the anode. On the other hand, a binaural monopolar GVS induces body sways in the sagittal plan [40]. The hyperpolarisation induced by the anodic electrodes placed on the mastoid bones generate body sways in the backward direction whereas the depolarisation induced by the inverted GVS design (i.e., cathodic electrodes placed on mastoids) induce body sways in the forward direction [38].

Numerous studies had analysed GVS effects on postural control in healthy or pathological participants [6,41–47], or in relation with aging [48–50]. Nevertheless, to our knowledge, few studies have analysed GVS effects, in relation to physical activity status by focusing on postural control [31,51]. More precisely, previous studies had focused on direction detection thresholds of passive self-motion in relation to sports [52] and the effects of sports activities on vestibulo-ocular reflex (VOR) [10,53–56]. Other authors focused on sports specific vestibular stimulation (e.g., head and body rotation) effects on static postural control [57]. From these previous studies, it is well established that the regular practice of physical and sport activities enhances CNS ability to reweight vestibular information according to the context/situation.

Concerning GVS, Balter et al. [51] showed that GVS effects were significantly lower for physically active participants (i.e. young professional gymnast) than control participants on postural balance. In addition, Yang et al. [58] also showed that experienced airplane pilots demonstrated less postural balance alteration to withstand GVS than control participants. However, Maitre et al. [31] found no difference between physically active participants (i.e. old sportswomen) and control participants in context of a GVS condition. Several factors may explain the differences between these studies. The currents characteristics of the GVS such as intensity and polarity could alter the results. The current intensity could be an important factor that may exhibit or not divergence between groups of difference physical status. Maitre et al. [31] construed that the GVS intensity would not be strong enough to generate any difference. Balter et al. [51] showed that postural differences with a current intensity of 2 mA. Yang et al. [58] also demonstrated differences with a maximal current intensity of 1.2 mA. Maitre et al. [31] demonstrated no differences with a current intensity of 1 mA. Moreover, no study had analysed the polarity effects on postural control in relation with physical activity. Yet, Lacour et al. [59] hypothesized a vestibular prevalence in healthy subject during GVS.

Regarding physical activity status, in order to take the analysis of postural behaviour in the context of GVS one step further, different current intensities and polarities should be studied. The aim was thus to compare the effects of GVS, with different intensities and polarities, on postural control for participants of different physical activity status. It would be interesting to choose an intensity slightly lower than Maitre et al. [31] (e.g. 0.5 mA) and an intensity slightly higher than Balter et al. [51] (e.g. 3 mA) in order to distinguish the intensity effects on postural control according to the physical activity status of participants. We hypothesized that the magnitude of the postural control impairment due to GVS effects would differ in accordance with physical activity status and in relation with the currents characteristics (current intensity and polarity). More precisely, for the higher GVS intensity at least, the postural control impairment would differ between individuals who practised sport (i.e., active participants) and individuals who did not practise sport (i.e. non-active participants). In addition, postural control impairment could differ according to the the GVS polarity used.

## Methods

Thirty-four healthy women, shown to be free from any neurological, motor and metabolic disorders after medical examination, participated in the study. Seventeen participants who practised sport (active group) and seventeen participants who did not practise sport (non-active group) were recruited. Age and anthropometrical data are presented in Table 1. This experimental procedure received the approval of the local committee for the protection of human participants (Comité de Protection des Personnes Sud-Ouest et Outre Mer I; approval number 2009-A00135-52) and all participants gave their written informed consent. After interviewing each subject, we included in the active group sport science students who have practised sports in competition (e.g. swimming, gymnastics, handball, basketball, athletics) at least at regional level, who trained three times per week (for 3 hours or more each week), in addition to physical and/or sport activities practised at sport science school (for 6 hours or more each week). We included in the non-active group persons who have not practised physical and/or sport activities for at least 3 years. Participants were restricted from stimulating substance consumption and strenuous exercises one day before the data collection.

**Table 1:** Mean (SD) median of the age and the anthropometrical data for the active and non-active groups.

	Active	Non-active	Wilcoxon-Mann-Whitney test
**Age (yr)**	20.5 (1.1) 20.7	20.0 (1.3) 19.7	NS
**Height (cm)**	164.8 (5.7) 165.0	162.3 (5.4) 163.0	NS
**Weight (kg)**	60.5 (7.1) 59.6	56.2 (9.2) 53.0	NS
**Foot size (cm)**	26.0 (0.9) 26.0	25.5 (0.8) 25.3	NS

Note. The median level significance differences are included in the table at the level of 5%. The Wilcoxon-Mann–Whitney test indicates significant group difference. NS: not signicant.

Participants were instructed to stand up (barefoot, bipodal position, outstretched legs, inter-malleolar distance of 9 cm, feet angle of 30° according to precise marks, arms along their trunk) on a force plateform (Techno Concept™, Cereste, France; 40 Hz frequency, 12 bit A/D conversion) with their eyes open fixed on a 4 cm^2^ target, 1.5 m in front of them at the height of their eyes.

They underwent five conditions, each lasting 20 seconds and separated by 2 minutes. To avoid the training effect between the different conditions, one trial was carried out to adapt the participants to the exercise prior to recording. They were tested in a reference condition (REF condition, i.e. quiet stance) and four sensory manipulation conditions. The main objective of these sensory manipulation conditions was to alter the vestibular information by means of GVS, which induces an asymmetry of vestibular activity [22].

A transmastoïdal GVS was delivered by a constant-current stimulator (Galvadyn2, Electronic Conseil, Gallargues le Montueux, France) through two disposable electrodes (T-Tracet, Contrôle-Graphique, Brie-Comte-Robert, France). The electrodes were placed over each mastoid process, for a bilateral bipolar design. To avoid any local noxious sensation, an anaesthetic gel was applied onto each mastoid process. Participants were measured in two current intensities and two different polarity conditions for each current intensity:

○ GVS-R (0.5 mA, 3 mA): an anode placed on the right mastoid process and a cathode placed on the left mastoid process.
○ GVS-L (0.5 mA, 3 mA): an anode placed on the left mastoid process and a cathode placed on the right mastoid process.

To avoid initial transients and anticipation behaviour recording at the onset of the sensory disturbance, each sensory manipulation was set up in a range of 5 seconds before the recording of postural sway data. The sensory stimulation was maintained throughout the entire duration of the data recording.

Posturowin software (Techno Concept™, Cereste, France) calculated the centre of foot pressure (COP) displacement parameters that characterise the postural behaviour. We use the COP velocity since it has been considered the measure with the highest reliability among trials [60,61]. The COP velocity is an indicator of the participant’s postural control and characterize the net neuromuscular activity required to maintain balance [60,61]. The smaller the COP velocity the better is the postural control. The COP velocity can be detailed on the medio-lateral axis in COP_X_ velocity (mm.s^-1^) and on the anterior-posterior axis in COP_Y_ velocity (mm.s^-1^). The absolute increases between REF and the other conditions were calculated for all the parameters:

Absoluteincrease=GVScondition−REF

Statistical analyses were performed with R statistical software. Age and anthropometrical data were compared using the non-parametric Wilcoxon-Mann-Whitney test to determine if there were differences between the two groups. The Friedman analysis of variance was performed to determine whether there were differences between the reference condition (REF) and the other conditions concerning all the parameters describing body sways. When significant treatment effect occurred, the pairwise Wilcoxon-Mann-Whitney post-hoc analyses with Holm-Bonferroni correction were used to test the difference among medians. The non-parametric Wilcoxon-Mann-Whitney test was also performed to determine if there were differences between the two groups concerning all the parameters describing body sways. Results were considered significant at the level of 5%.

## Results

Means and standard deviations are presented with the medians in Tables 1, 2 and 3. The results concerning age and anthropometrical data are presented in Table 1. The centre of foot pressure velocity displacements on the X and Y axis are presented in Tables 2 and 3 respectively, with their absolute increases.

**Table 2:** Centre of foot pressure velocity displacements on the X (mediolateral direction) axes and their absolute increases [mean (standard deviation) median], in reference and GVS conditions for the active and non-active groups.

	Active	Non-active	Wilcoxon-Mann-Whitney test
REF	3.93 (0.84) 4.00	4.36 (0.98) 4.17	NS
0.5 mA—R	4.31 (0.93) 4.41	4.73 (1.39) 4.20	NS
3 mA—R	4.72 (1.81) 4.31	6.91 (2.67) 6.28†‡	W = 224, p = 0.005
Friedman ANOVA	NS	χ^2^(2) = 14.9; p = 0.0006	
0.5 mA—L	3.91 (0.97) 3.86	4.8 (1.12) 4.56	W = 207, p = 0.03
3 mA—L	4.66 (1.35) 4.33	6.16 (2.53) 5.72†‡	W = 203.5, p = 0.04
Friedman ANOVA	NS	χ^2^(2) = 7.9; p = 0.02	
Absolute increases			
0.5 mA—R	0.38 (0.82) 0.55	0.37 (1.49) -0.04	NS
3 mA—R	0.79 (1.77) 0.67	2.55 (2.29) 2.45	W = 219, p = 0.01
0.5 mA—L	-0.02 (0.91) -0.09	0.44 (1.20) 0.55	NS
3 mA—L	0.73 (1.26) 0.50	1.80 (2.04) 1.35	NS

Note. The median level significance differences are included in the table at the level of 5%. The Wilcoxon-Mann-Whitney test indicates significant group difference. The Friedman analysis of variance indicates significant condition difference.

† indicates significant post hoc analysis difference from REF condition.

‡ indicates significant post hoc analysis difference from the 0.5 mA condition (same polarity). NS: not signicant.

**Table 3:** Centre of foot pressure velocity displacements on the Y (anteroposterior direction) axes and their absolute increases [mean (standard deviation) median], in reference and GVS conditions for the active and non-active groups.

	Active	Non-active	Wilcoxon-Mann–Whitney test
REF	4.50 (1.03) 4.28	4.97 (1.13) 4.80	NS
0.5 mA—R	4.92 (0.92) 5.29	5.56 (1.65) 5.38	NS
3 mA—R	5.59 (1.63) 5.72	7.99 (3.46) 7.05†‡	W = 209, p = 0.03
Friedman ANOVA	NS	χ^2^(2) = 14.6; p = 0.0007	
0.5 mA—L	4.79 (1.41) 4.40	5.41 (1.55) 5.34	NS
3 mA—L	5.67 (1.44) 5.51	7.57 (3.22) 6.45†‡	NS
Friedman ANOVA	NS	χ^2^(2) = 20.6; p = 0.00003	
Absolute increases			
0.5 mA—R	0.43 (0.92) 0.43	0.59 (1.46) 0.05	NS
3 mA—R	1.09 (1.60) 0.81	3.02 (3.08) 1.99	W = 207, p = 0.03
0.5 mA—L	0.29 (1.08) 0.42	0.44 (1.34) 0.31	NS
3 mA—L	1.18 (1.49) 0.93	2.59 (2.49) 1.61	NS

Note. The median level significance differences are included in the table at the level of 5%. The Wilcoxon-Mann-Whitney test indicates significant group difference. The Friedman analysis of variance indicates significant condition difference.

† indicates significant post hoc analysis difference from REF condition.

‡ indicates significant post hoc analysis difference from the 0.5 mA condition (same polarity). NS: not signicant.

Age and Anthropometrical data results indicated no difference between the two groups concerning age, height, weight and foot size (Table 1).

Postural control parameters results indicated that there was no significant difference between the two groups in the initial condition (REF). The main results indicated that GVS altered postural control for the non-active group but not for the active group (Tables 2 and 3). The COP_X_ and the COP_Y_ velocity increased for the 3 mA-R and the 3 mA-L conditions for the non-active group compared with the initial reference condition (REF). In addition, the COP_X_ and the COP_Y_ velocities increased significantly more for the non-active group than the active group only for the 3 mA-R condition (absolute increases, Tables 2 and 3).

## Discussion

The aim of this study was to compare postural behaviour to different GVS intensities and polarities between active participants and non-active participants. Our hypothesis was confirmed since the results indicate that the main difference between the two groups of participants arise from the highest GVS intensity. The main differences between the two groups appeared when the anode was placed on the right mastoid process and the cathode was placed on the left mastoid process (i.e. GVS-R design). Moreover, the regular practice of sports activities counteracts postural control disruption of GVS for all current intensities and polarities. The physically active group demonstrated better postural control than the non-active group for the more intense vestibular manipulation

In the present study the COP_X_ and the COP_Y_ velocities increased significantly more for the non-active group than the active group at 3 mA (for GVS-R and GVS-L). One can suggest that GVS had less effect on postural control for the active group than the non-active group thanks to their vestibular system adaptation and/or habituation capabilities to the stimulus and their ability to reweight sensory information. In this study, the most disruptive postural effects of GVS involve the highest GVS intensity which require important compensatory postural mechanisms in order to withstand the stimulation. Unlike the lowest intensity, the highest GVS intensity evidenced different postural abilities. Hence, it would exist a minimal GVS intensity to highlight different postural abilities between individuals which would be largely depends on the individuals’ postural skills.

The active group likely took advantage from their experience of vestibular stimulation (i.e. rotation and translation, acceleration and deceleration) to decrease the GVS effects on postural control. It has been shown that the repetition of the situation that provoke vestibular perturbation have been successfully used to alleviate disorienting effects of the situation (e.g., vestibular habituation exercises in context of benign paroxysmal positional vertigo) [62]. Sports activities involve head motion and sudden direction changes (including rough deceleration of the head that gives erroneous vestibular information of an opposite movement to the initial movement direction), which strongly stimulate the vestibular function. Previous studies have in fact demonstrated that the repeated stimulation of the vestibular system (e.g. acceleration and deceleration) may habituate the individuals to the stimulus [10,12,53,55]. This habituation enables a decrease of the stimulus response (e.g. duration and amplitude of the disorientation). The regular practice of sports activities such as ballet dancing [55] and gymnastics [10] generates habituation to the stimulus of the vestibular function.

The CNS interprets bipolar GVS signal as a real head movement (i.e. head rotation) in space arising from an unplanned body movement [30]. The balance system triggers, at least in its early stage, an inappropriate postural response since GVS induce a fictitious vestibular signal [30]. Subsequently, to withstand this erroneous vestibular signal the CNS generates adaptive postural adjustments to compensate disruptive postural effects of the GVS [42]. The results of the present study corroborate previous research which have indicated that the repeated mechanical stimulation of the vestibular system may serve to alleviate the postural disrupting effect of GVS on postural control [51,58]. Balter et al. [51] showed that gymnasts demonstrated better postural control than control participants when they were subjected to GVS. They suggested that the postural control of elite gymnasts was enhanced through motor training. Furthermore, Yang et al. [58] showed that trained airplane pilots have better postural control than control participants when they were subjected to GVS. These authors suggested that the pilots’ vestibular habituation training enables them to withstand greater vestibular stimulation by improving their ability to suppress vestibular illusions. Thus, in the present study, the repeated stimulation of the vestibular system induced by the regular practice of sport activities, such as e.g., tumble turn in natation, somersault in gymnastic, brutal head rotation in collective sports, might have trained the active group to withstand the vestibular illusion of motion and might have reduced its influence on postural control. It may also be noted that even a short-term vestibular training (i.e. 20 hours of flight training) is sufficient to change vestibular function [25]. Lee et al. [25] suggested that this change originate from a plastic adaptive process rather from habituation. Adaptation and habituation mechanisms might contribute to reduce the disturbing effects of GVS. Nevertheless, these mechanisms are strongly dependent of the task [23,27,56] and so cannot be the only factors that explain the better postural behaviour of the active group compared to the no active group in context of vestibular stimulation.

The better postural performance achieved by the active group compared to the non-active group could also be linked to the ability of members of the active group to take advantage of other available sensory information. The active group may have developed better use of the non-disrupted sensory afferents (i.e. visual, proprioceptive and cutaneous sensory information) than the non-active group in the context of vestibular illusion. The active group was able to counteract GVS effects on postural control. No significant difference was found between the reference condition and the GVS conditions for the active group. Inversely, the non-active group was not able to counteract the influence of the 3 mA GVS effects on postural control. In the present study, we have to take into account the availability of the visual sensory system. Visual afferents are crucial elements, which enable the influence of vestibular stimulation to be reduced [31,63,64]. Moreover, Teramoto et al. [55] have suggested that dancers are able to acquire skill in controlling posture mainly by using visual input in rotational movements as a result of their training. We can thus suggest that the physically active group showed no effect as a result of vestibular stimulation partly because of the availability of visual cues. Moreover, the non-active group may have benefited from the availability of visual afferents only to compensate the postural effects of the 0.5 mA GVS conditions. As suggested above, the non-active group was not able to take advantage of the availability of the visual sensory system as effectively as the active group. This probably indicates the existence of a stimulation threshold value of intensity, which distinguishes differences in postural ability between the active group and the non-active group.

Although visual information has greater influence than vestibular information during a visuo-vestibular conflict, the CNS may privilege an additional appropriate input in order to overcome the sensory conflict [65]. It has been demonstrated that physical and sports activities may generate specific postural improvement [33,34]. Balance training may lead to task-specific neural adaptations at the spinal and supraspinal levels that modulate spinal reflex excitability, which enhance postural control [32]. As suggested by Hrysomallis [33], physically active participants group may become more skilled at focusing on and attending to important sensory cues with training, thereby producing refined motor responses. This sensory conflict can be resolved by an adaptive response in which the participant suppresses inaccurate inputs and selects more accurate sensory inputs in order to generate appropriate motor responses and postural strategies. This could be characterised by an under reliance on vestibular and visual inputs and an increased dependence on the somatosensory system for the maintenance of balance [6,65–68]. Hence, one can also suggest that the active group may have used the proprioceptive and the plantar cutaneous afferents more effectively than the non-active group in the context of vestibular perturbation.

It is interesting to note that the major differences of the COP_X_ and COP_Y_ velocities between the two groups, at the 3 mA current intensity, exist only for the GVS design anode placed on the right mastoid process and cathode placed on the left mastoid process (i.e. GVS-R). This result might be linked to a more important postural effect of the GVS-R than the GVS-L. Different magnitude effects of GVS, depending on the electrode polarities, have been observed [59]. These authors hypothesized a vestibular prevalence in healthy subject during GVS. In addition, it has been demonstrated that GVS effects may be altered by the lateralization [69]. Otherwise, it could be construed that physically active participants would have developed a preferential habituation to vestibular illusion of rotation in a specific direction. Indeed, sports activities may generate task specific adaptation [32–34]. Quarck and Denise [10] have suggested that repeated body rotation in a particular direction resulting from sport (gymnastic in this study) may generate vestibular asymmetry. More precisely, this asymmetry may result in a preferential rotation direction in sports. With regard to these elements it would appear that there are differences between the GVS- R and the GVS-L postural effects. Nevertheless, the statistical comparison of the COP velocities between the two GVS designs at 3 mA (GVS-R vs GVS-L) failed to reveal any significant difference for the two groups in terms of velocity and absolute increase (results calculated but not presented).

Another intriguing result indicates that there is no difference between the active group and the non-active group in the reference condition. This result is not supported by the literature. Previous studies have demonstrated that, in general, sports practitioners sway less than control participants in unperturbed stance (see Kiers et al. [34] for review). However, several aspects of the literature provide some basis for explaining this postural behaviour. Firstly, the reference condition may not have challenged postural control enough to produce evidence of differences between the active group and the non-active group. Indeed, the analysis of body sway in unperturbed bipedal stance appears to have limited sensitivity for detecting subtle differences between groups of healthy people [34]. In addition, Kiers et al. [34] have also suggested that a more challenging postural condition (e.g. standing on foam or unipedal stance) should be used to demonstrate differences between healthy people. The difference between the two groups was probably not significant because the postural task was too easy for the purposes of determining differences in postural ability [70]. Secondly, most of the participants in the active group practise physical activities that do not specifically require static postural control (i.e. swimming, handball, basketball, athletics) in comparison to the non-active group. Furthermore, sports activities generate specific postural adaptation [32,33], which implies that experts in sports may be more efficient in conditions that are consistent with their prior experience and training [71]. Thus, as suggested by Paillard et al. [70], the characteristics of the postural task (in the REF condition) might not be specific enough to enable the active group to demonstrate better postural control than the non-active group.

## Limitations of the Study

There are several limitations in the present study. In a context of a sensory alteration, the CNS use sensory reweighting to adjust the relative contribution of afferents emanating from the visual, vestibular and somatosensory systems to maintain an upright stance [6]. Nevertheless, in healthy individuals the contribution of each sensory system may differ between individuals [44,72]. For example, several authors have reported that some individuals use visual information to improve their balance whereas others do not [73,74]. In addition, physical and sports activities may also generate dependence to a sensory system depends on the type of sport practised [33,34]. Hence, since the participants included in the active group practice different type of physical activity, we cannot exclude that they react differently to the vestibular disruption (i.e. GVS). In this group, one of the participants might have dragged the population. Otherwise, the postural effects of the GVS are not only limited to the GVS intensity. They also depend on the GVS design (i.e., monaural, binaural; monopolar, bipolar), the stimulation duration and the current characteristics (e.g., biphasic, ramp, sinusoidal). Hence, these parameters may have influenced the results.

## Conclusion

In this study, the main difference between the two groups of participants arise from the highest GVS intensity (i.e. 3 mA, GVS-R design). The active group was composed of individuals who practise sport at regional or national level, which indicates an important difference of level when compared to the non-active group. They practise sports that strongly stimulate the vestibular system. It may be that, with experience, the active participants develop their ability to reduce the influence of inaccurate vestibular signals on postural regulation. The repeated vestibular stimulation induced by the regular practice of physical activity may have reduced the GVS effect on postural control. In addition, it could be that the active participants switch faster between sensory inputs, which suggests that they have better central integration and ability to identify the most relevant sensory input [51]. Such a strategy would allow the conflicting visual and vestibular inputs to be ignored due to the provision of additional appropriate inputs (i.e. somatosensory inputs) [6,65–68]. Few studies have explored the GVS technique to understand how physical activity influences postural control. This study supports previous observations by Balter et al. [51] and Yang et al. [58]. The magnitude of the postural responses to GVS was significantly different between the active group and the non-active group. If the GVS intensity is correctly calibrated, the GVS could be a relevant tool to determine the postural abilities of sports practitioners. To take the analysis of postural behaviour in the context of GVS in relation to physical activity one step further it would be interesting to analyse different sport activities.

## Supporting Information

S1 Data Data of centre of foot pressure velocity displacements on the mediolateral and anteroposterior directions. (XLSX)
